# Study protocol for a randomised pragmatic trial comparing the clinical and cost effectiveness of lithium and quetiapine augmentation in treatment resistant depression (the LQD study)

**DOI:** 10.1186/s12888-017-1393-0

**Published:** 2017-06-26

**Authors:** L. Marwood, R. Taylor, K. Goldsmith, R. Romeo, R. Holland, A. Pickles, J. Hutchinson, D. Dietch, A. Cipriani, R. Nair, M.-J. Attenburrow, A. H. Young, J. Geddes, R. H. McAllister-Williams, A. J. Cleare

**Affiliations:** 1 Centre for Affective Disorders, Department of Psychological Medicine, Institute of Psychiatry, Psychology & Neuroscience, King’s College London, London, UK; 2 South London and Maudsley NHS Foundation Trust, London, UK; 3 Biostatistics & Health Informatics Department, Institute of Psychiatry, Psychology & Neuroscience, King’s College London, London, UK; 4 Health Services and Population Research, Institute of Psychiatry, Psychology & Neuroscience, King’s College London, London, UK; 5 Northumberland, Tyne and Wear NHS Foundation Trust, Newcastle upon Tyne, UK; 6 Institute of Neuroscience, Newcastle University, Newcastle upon Tyne, UK; 7 Lonsdale Medical Centre, London, UK; 8 Department of Psychiatry, University of Oxford, Oxford, UK; 9 Oxford Health NHS Foundation Trust, Oxford, UK; 10 Tees, Esk and Wear Valleys NHS Foundation Trust, Darlington, UK

**Keywords:** Treatment resistant depression, Lithium, Quetiapine, Pragmatic, Randomised clinical trial, Longitudinal, Open-label, Multi-centre, Augmentation, Superiority design

## Abstract

**Background:**

Approximately 30–50% of patients with major depressive disorder can be classed as treatment resistant, widely defined as a failure to respond to two or more adequate trials of antidepressants in the current episode. Treatment resistant depression is associated with a poorer prognosis and higher mortality rates. One treatment option is to augment an existing antidepressant with a second agent. Lithium and the atypical antipsychotic quetiapine are two such add-on therapies and are currently recommended as first line options for treatment resistant depression. However, whilst neither treatment has been established as superior to the other in short-term studies, they have yet to be compared head-to-head in longer term studies, or with a superiority design in this patient group.

**Methods:**

The **L**ithium versus **Q**uetiapine in **D**epression (**LQD**) study is a parallel group, multi-centre, pragmatic, open-label, patient randomised clinical trial designed to address this gap in knowledge. The study will compare the clinical and cost effectiveness of the decision to prescribe lithium or quetiapine add-on therapy to antidepressant medication for patients with treatment resistant depression. Patients will be randomised 1:1 and followed up over 12 months, with the hypothesis being that quetiapine will be superior to lithium. The primary outcomes will be: (1) time to all-cause treatment discontinuation over one year, and (2) self-rated depression symptoms rated weekly for one year via the Quick Inventory of Depressive Symptomatology. Other outcomes will include between group differences in response and remission rates, quality of life, social functioning, cost-effectiveness and the frequency of serious adverse events and side effects.

**Discussion:**

The trial aims to help shape the treatment pathway for patients with treatment resistant depression, by determining whether the decision to prescribe quetiapine is superior to lithium. Strengths of the study include its pragmatic superiority design, broad inclusion criteria (external validity) and longer follow up than previous studies.

**Trial registration:**

ISRCTN registry: ISRCTN16387615, registered 28 February 2016. ClinicalTrials.gov: NCT03004521, registered 17 November 2016.

## Background

Major depressive disorder (MDD) is a highly prevalent and disabling illness requiring effective treatment in order to reduce symptom severity and improve quality of life [1, 2]. Current clinical guidelines recommend the use of antidepressant medication in cases of moderate to severe MDD [3]. However, approximately 30–50% of patients fail to adequately respond to both first and second line antidepressant treatment trials, and can therefore be described as treatment resistant [3–5]. Existing evidence clearly indicates that the prognoses for patients with treatment resistant MDD (TRD) can be improved with adequate multimodal and/or successive treatment trials [4, 6]. Given that TRD is associated with a generally poorer prognosis, higher mortality rates and higher healthcare utilisation costs than MDD alone [7, 8], the importance of ensuring that the most effective available medication is fully utilised in clinical practice is clear.

Treatment options for patients with TRD include increasing the dose of a patient’s existing antidepressant, switching to another antidepressant (same or different class), or augmenting the existing antidepressant medication with a second agent, for example an additional antidepressant, mood stabiliser, or antipsychotic medication [3]. Increases in dosage have been associated with increased efficacy for some antidepressants [7, 9–11] but not for others [12–15]. Switching to an alternative antidepressant is generally suggested in cases where a patient has either made no response, or is not tolerating their current medication [3], but remission rates to third or fourth line antidepressant treatments are in the order of just 10–15% [4]. For TRD patients in whom there has been a partial response to current antidepressant treatment, augmentation is usually recommended [3]. However, beyond these general principles, there remains much uncertainty about when to switch and when to augment treatments.

One augmentation strategy is to prescribe a combination of antidepressant medications. However, recent evidence from a large randomised controlled trial indicated that this approach is no more effective than antidepressant monotherapy, questioning its appropriateness [16]. The use of lithium or atypical second-generation antipsychotic augmentation is an approach supported by several recent meta-analyses [17, 18]. This is reflected in the National Institute of Clinical Excellence (NICE) and the British Association for Psychopharmacology (BAP) treatment guidelines, which emphasise that add-on treatments are a standard treatment pathway for TRD, and include lithium and newer atypical antipsychotics, such as quetiapine, aripiprazole, olanzapine and risperidone, as first line options [3, 19].

Despite these recommendations, few studies have compared the efficacy of lithium versus atypical antipsychotic augmentation head-to-head [3, 20, 21]. The largest and arguably best study to date compared lithium and quetiapine extended release (XR) over just 6 weeks, and found quetiapine to be non-inferior to lithium add-on therapy in patients with TRD [20]. Lithium versus quetiapine augmentation has also been compared in a pilot study of 20 patients, which reported significant reduction in depressive symptoms in both groups, but a greater decrease in Hamilton Depression Rating Scale (HDRS) scores from day 14 onwards in the quetiapine group [21]. However, neither of these comparisons have included a long term follow up, which is needed to ensure that the most effective treatment option is fully utilised in clinical practice, particularly given the chronic nature of TRD, the associated high rates of relapse [8], and the fluctuating nature of depressive symptoms (even in those responding to acute phase treatment) [16]. Clinical guidelines also advise that responders should continue taking the effective medication for at least 6–12 months [3, 19]. Therefore a clinical trial comparing lithium and quetiapine augmentation, with frequent assessment of symptoms and 12 month follow up is clearly warranted.

In the present study, lithium augmentation will comprise one treatment arm, as it is the first-choice add-on treatment for patients with TRD according to the World Federation of Societies of Biological Psychiatry Task Force [22], and a first line treatment option recommended by NICE and BAP [3, 19]. Quetiapine was selected as the atypical antipsychotic comparator, as there is strong evidence for the efficacy of quetiapine augmentation versus placebo [23–25], and it is currently licenced for marketing as an add-on treatment for TRD in the UK in its XR formulation [19]. Although NICE does support the use of other atypical antipsychotics (aripiprazole, risperidone and olanzapine) as add-on treatments in TRD, and there is some evidential support for their use [17, 26], quetiapine is the only atypical antipsychotic to have been previously studied under trial conditions in a head-to-head comparison with lithium, meaning at least comparable short-term effectiveness between the two treatments under trial conditions is known [20]. While this study by Bauer and colleagues reported quetiapine to be non-inferior to lithium over a six week treatment course, there was also some indication that quetiapine may be more clinically effective than lithium. This included lower depression severity scores (Montgomery-Åsberg Depression Rating Scale, MÅDRS), reported as early as day 4 of treatment, and less sleep disturbance [20]. Additionally, a network meta-analysis comparing augmentation agents in TRD found lithium and quetiapine to both be more effective than a placebo, but indicated that the efficacy of quetiapine was more robust than lithium [27].

### Objectives and hypothesis

This study aims to determine whether the decision to prescribe quetiapine is more clinically and cost effective than the decision to prescribe lithium over a longer term in a randomised clinical trial powered to detect superiority. We propose a 12 month follow up period, hypothesising that quetiapine will be superior to lithium in terms of our primary outcomes: time to all-cause treatment discontinuation and average depressive symptom burden over 12 months.

We hope to help shape a modified treatment pathway for TRD in which one augmenter is preferential to the other. However, if quetiapine is not found to be clinically superior based on our primary and secondary outcomes, the results of the cost effectiveness analysis, or individual level predictive factors, may determine the most appropriate choice for each patient.

The trial will recruit patients with TRD: defined as the failure to adequately respond to two or more therapeutic trials of antidepressant treatments in the current episode (at adequate dose and duration) [3, 8]. This definition of treatment resistance is in line with the point at which treatment augmentation is recommended in clinical practice [3] and at which the evidence suggests clinical equipoise between the two treatments [20].

This protocol paper is consistent with SPIRIT (Standard Protocol Items: Recommendations for Interventional Trials) 2013 recommendations [28] (checklist attached).

## Methods/design

### Study design

LQD is a phase 4, 12-month, parallel group, multi-centre, patient 1:1 randomised, pragmatic, open-label, superiority trial, comparing the clinical and cost-effectiveness of the decision to prescribe lithium versus quetiapine as add-on treatment to antidepressant medication in patients with TRD.

### Participants

We will recruit participants who fulfil the following inclusion criteria:

1. Under the care of a GP and/or adult mental health services
2. Current episode of depression meeting Diagnostic and Statistical Manual of Mental Health Disorders – Fifth Edition (DSM-5) [29] criteria for MDD (single or recurrent episode) assessed using the Mini International Neuropsychiatric Interview, Version 7 (MINI 7.0) [30]
3. HDRS - 17 item [31] score ≥ 14 at screening
4. Any gender and aged 18 years or over
5. TRD [3, 32]: defined as failing to adequately respond to at least two antidepressant therapies, prescribed for at least 6 weeks at minimum therapeutic dose, as determined by the Maudsley Prescribing Guidelines (MPG) and/or British National Formulary (BNF) [33, 34]
6. Current antidepressant treatment has remained unchanged for ≥6 weeks
7. Provision of written, informed consent

Exclusion criteria for participants are:

1. Diagnosis of bipolar disorder (defined as meeting DSM-5 criteria for bipolar 1 or 2) on the MINI 7.0
2. Diagnosis of current psychosis
3. Use of lithium or quetiapine during current depressive episode
4. Ongoing use of another atypical antipsychotic
5. Known contraindication to use of either lithium or quetiapine
6. Currently participating in another clinical trial of an investigational medical product
7. Insufficient degree of comprehension or attention to be able to engage in trial procedures
8. Pregnancy, actively trying for pregnancy, or breastfeeding

### Recruitment procedure and assessments

Participant recruitment will take place across three of the UK National Institute of Health Research (NIHR) Clinical Research Network hubs: South London, the North East and North Cumbria, and Thames Valley and South Midlands. Recruitment is underway at four sites within these hubs: the South London and Maudsley National Health Service (NHS) Foundation Trust (SLaM); Northumberland, Tyne and Wear NHS Foundation Trust (NTW); Oxford Health NHS Foundation Trust (OHT); and Tees, Esk and Wear Valleys NHS Foundation Trust (TEWV).

Potential participants will be identified primarily at routine secondary care clinic appointments or via Consent for Contact initiatives within the trusts (e.g. http://www.slam.nhs.uk/research/patient-involvement/current-opportunities/consent-for-contact) alongside advertisements, and via Participant Identification Centres including primary care and external UK NHS trusts to enhance recruitment.

### Randomisation and blinding

The study will randomise individual participants 1:1 to the decision to prescribe lithium or quetiapine, stratified by recruiting region (London, Oxfordshire, or North East England), depression severity (HDRS-17), and TRD severity (failure of two / three or more antidepressant treatments in the current episode), using block randomisation with randomly varying block size. Randomisation will be conducted by a trial researcher using an independent online service provided by UK Clinical Research Collaboration registered King’s Clinical Trial Unit, with unique patient identification numbers generated by the InferMed MACRO system.

This is an unblinded study, whereby treating clinicians, trial researchers, and patients will be aware of the treatment allocation for patients where applicable to their role. In order to reduce the potential for assessor bias, the following clinician-rated assessments will be completed at follow up visits by an assessor who is blind to the patient’s medication (face-to-face or via phone): 1) MÅDRS [35], and 2) Clinical Global Impressions scale (CGI) [36]. Trained trial researchers and clinicians may perform blinded assessments, but must not have access to the trial database for the relevant site to reduce the likelihood of unblinding (access to the trial database is granted on a site by site basis, dependent on role). The blinded assessor will remind the participant not to reveal treatment allocation at the start of the assessment and will confirm they remained blinded to treatment allocation throughout the assessment.

### Primary outcomes

- Difference in time to all-cause treatment discontinuation over 12 months post-randomisation between lithium and quetiapine using survival analysis methods, with time being between the first prescription and discontinuation of the medication to which the patient was randomised.
- Longitudinal Depression Severity: the difference in the area under the curve between lithium and quetiapine over 12 months post-randomisation in the self-rated Quick-Inventory of Depressive Symptomatology (QIDS-SR) [37], assessed weekly via the True Colours system (www.truecolours.nhs.uk), using a linear mixed model, covarying for baseline QIDS-SR score.

### Secondary outcomes

The secondary outcomes will be measured at 8 and 52 weeks, and differences between lithium and quetiapine assessed at both time points. Continuous secondary outcomes measured over time will use appropriate generalised linear models, covarying by baseline score where applicable. Where there are repeated measures of a continuous variable, the analysis will be set in the mixed model framework. Time to event variables will be analysed using survival methods. The secondary outcomes are as follows:

- Clinician-rated depression severity (continuous total score on the MÅDRS).
- Clinician-rated response and remission rates (defined as proportion with ≥50% reduction in baseline MÅDRS total score and MÅDRS total score ≤ 10, respectively).
- Global improvement (proportion of patients with a CGI-improvement score of ‘much’ or ‘very much improved’).
- Health-related quality of life (summary index EuroQol-5D, EQ-5D score [38]).
- Social functioning (continuous total Work & Social Adjustment Scale (WSAS) score [39]).
- Self-report adherence to treatment (5-item Medication Adherence Report (MARS-5) continuous total scores and exploratory cut-offs categorising patients as either adherent or non-adherent [40]).
- Physical health parameters: weight (kg) and blood pressure (mmHg).
- Time to commencement of trial medication from randomisation (and number of patients commencing therapy in each arm).
- Time to uptake of a new intervention for depression from randomisation (pharmacological or non-pharmacological) over 12 months (and proportion of patients in each arm receiving new interventions).
- Side Effects (continuous total score on the Patient Rated Inventory of Side Effects (PRISE) [41]).
- Frequency of Serious Adverse Events (SAEs).

Secondary outcomes, except for SAEs, will be adjusted for multiple comparisons. SAEs will be tabulated as number of SAEs in each group, and number of people reporting an SAE in each group over 12 months. We will also summarise the reasons for clinicians not prescribing and patients not commencing the medication they are randomised to, reasons for treatment discontinuation, the number of withdrawals, and the reasons for withdrawals from the trial. However, we will not test the difference between the groups on these variables statistically.

### Additional outcomes

For a full list of tertiary and ancillary trial outcomes please visit clinicaltrials.gov. Two key ancillary outcomes are detailed below:

- **Cost-effectiveness**: This data will be gathered from the Client Service Receipt Inventory (CSRI) [42], modified for TRD, asked at baseline, 8, 26, and 52 week study visits. Cost-effectiveness analyses will be conducted from a health and social care, and a societal perspective. This will include trial and concomitant medication costs, healthcare costs (including hospital, community-based, social and primary care services), costs to statutory and non-statutory services, impact on caregivers and families, and days off work due to health problems.
- **Predictors of treatment response**: for example, baseline staging of treatment resistance (Maudsley Staging Method, MSM [32]), depression characteristics (e.g. severity (HDRS [31]), chronicity, family history, recurrence (MINI 7.0), psychiatric comorbidity (MINI 7.0), subtype (e.g. typical versus atypical, clinician rated Inventory of Depressive Symptomatology (IDS-C) [43]), personality (Standard Assessment of Personality [44]), type of antidepressant (e.g. selective serotonin reuptake inhibitor (SSRI) vs. non-SSRI), hypomanic symptoms (16-item hypomanic checklist (HCL-16) [45] at baseline) and sociodemographic factors (e.g. sex, age, ethnicity, body mass index). Briefly, the analysis would build a predictive model of response using an appropriate variable selection procedure (i.e. manual forward stepwise regression).

### Interventions

As this is a pragmatic study, trial medications will be prescribed open-label and no measures will be taken to intervene with patients’ medication adherence. Trial clinicians will be advised to keep patients’ existing antidepressant treatment(s) at a stable dose within the therapeutic range, as defined in the MPG and BNF [33, 34]. The following recommendations for titration and dosing of each of the two treatment arms will be provided, in line with current best practice guidelines [33, 34]. However failure to adhere to these guidelines will not constitute a protocol deviation:

- *Lithium arm:* lithium carbonate/citrate, added on to the current antidepressant. The dose should be adjusted to achieve a serum-lithium concentration of 0.4–1.0 mmol/l 12 h after a dose. Serum level checks should be performed on days 4–7 of treatment and be repeated every week until dosage has remained constant for 4 weeks aiming for an optimal therapeutic plasma level of 0.6–1.0 mmol/l [22, 33].
- *Quetiapine arm:* quetiapine fumarate (XR or immediate release formulation) added on to the current antidepressant, taken once daily before bedtime. Recommended dose titration: 50 mg on days 1 and 2 and 150 mg on day 3, aiming for a dose of 300 mg/day by week 2, if tolerated. Thereafter, flexible dosing will follow in the range 150–300 mg/day according to tolerance (as per Bauer et al. 2013) [23]. In elderly patients (>65 years old), the dose titration protocol should be: 50 mg/day on days 1–3, increasing to 100 mg/day on day 4, 150 mg/day on day 8 and 300 mg/day not before day 22 of treatment, if required.

Dosing regimens may need to be altered in the case of concomitant administration of drugs that interact with either quetiapine or lithium.

The initial prescription of a study medication and any essential pre-prescription safety checks must be overseen by a trial clinician. This is followed by a fully flexible continuation phase for up to 52 weeks post-randomisation, in which treatment is shared between primary and secondary care as appropriate according to standard NHS practice in the relevant trust.

In this trial, patients will be randomised to *the decision* to prescribe either lithium or quetiapine. This means that in each case, the treating trial clinician will decide whether or not to prescribe the medication that the patient has been randomised to, according to their clinical judgement. It is therefore possible that a small proportion of randomised patients will not receive the medication that they are allocated to. This may be due to a previously unknown contraindication to the study medication arising during the pre-prescribing safety checks (exclusion criteria for trial entry is *known* contraindications to lithium or quetiapine only). The required safety checks are: electrocardiogram, if clinically indicated (both treatment arms) and pre-lithium blood tests (renal function, thyroid function, full blood count, serum calcium). These tests must be conducted prior to prescription, unless they have been completed within a sufficiently recent period according to the prescribing clinician’s judgement.

All patients will be followed up for 12 months, regardless of medication status. It is expected that a significant proportion of patients will fail to respond or tolerate the medication and will terminate treatment before 12 months. Treatment may also be extended beyond the follow up period at the discretion of the patient’s clinician. All concomitant pharmacological and non-pharmacological interventions are permitted throughout the duration of the trial and will be recorded by the trial team.

### Assessments

**Fig. 1:**
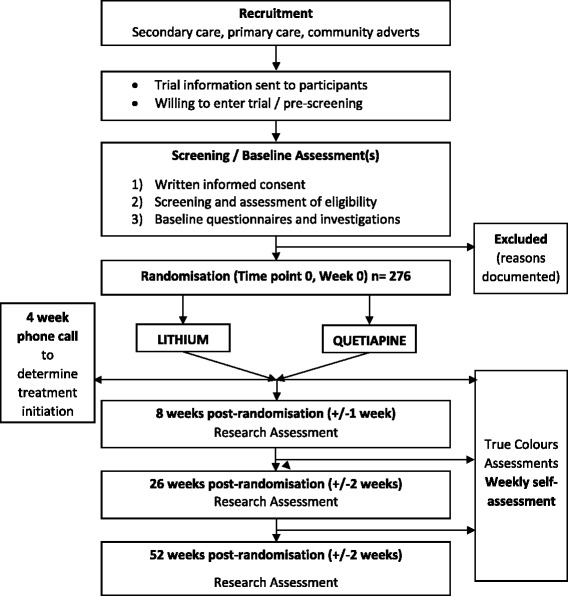
Flow Chart of Trial Procedures

All assessments, data entry and randomisation will be conducted by trained trial researchers with oversight from the clinical chief (AJC) and principal investigators (AHY, RHMW, JG, RN). See Table 1 for details of all study measures and Fig. 1 for a flow chart of trial procedures.

**Table 1:** Summary of measures

Time point	Screening	Baseline (Week 0)	Follow up			
			Weekly Assessments Weeks 0–52	Week 8 (+ − 1 weeks)	Week 26 (+ − 2 weeks)	Week 52 (+ − 2 weeks)
Written informed consent	√					
Assessment of eligibility	√					
MINI 7.0 to confirm MDD and other comorbid axis 1 disorders	√					
Assessment of depression severity (HDRS-17)	√					
Assessment of medication history in current depressive episode	√					
Sociodemographic / Psychiatric and Medical History (including MSM)	√					
Assessment of concomitant medication and non-pharmacological therapies	√	√		√	√	√
Clinician-rated assessment of clinical symptoms (MÅDRS, CGI)		√		√	√	√
Randomisation		√				
Clinician-rated depression severity (including subtype: IDS-C)^b^		√		√		
Hypomanic checklist (HCL-16)^b^		√				
Assessment of side effects (FIBSER^b^ and PRISE)				√	√	√
Assessment of quality of life (EQ-5D)		√		√	√	√
Assessment of cognition (THINC-it^a, b^ and DSCT^b^)		√		√	√	√
Weekly True Colours self-rated measures: QID-SR, WSAS, and trial medication status		√	√	√	√	√
Self-Rated clinical measures (ASRM^b^, Maudsley VAS measures^b^, GAD-7^b^, SAPAS^b^)		√		√	√	√
Assessment of costs (CSRI^b^ and employment status^b^)		√		√	√	√
Treatment satisfaction (TSQM^b^)				√	√	√
Adherence (baseline to antidepressant^b^, follow up to trial medication)		√		√	√	√
Qualitative assessment of patient experience of True Colours^a, b^				√	or √	or √
Physical health (weight, height, blood pressure, pulse rate, waist circumference)		√		√	√	√
Blood tests (FBC, U&Es, LFTs, TFT, glucose, lipids, calcium)^a, b^		√		√		√
Lithium and quetiapine serum levels^a, b^				√		√
BioResource genetic/cortisol/cytokine sample collection^a, b^		√		√		√

*MINI 7.0* Mini International Neuropsychiatric Interview, Version 7.0, *MDD* major depressive disorder, *HDRS* Hamilton Depression Rating Scale – 17 items, *MSM* Maudsley Staging Method, *MÅDRS* Montgomery-Åsberg Depression Rating Scale, *CGI* Clinical Global Impressions, *IDS-C* Inventory of Depressive Symptomatology – Clinician Rated, *HCL-16* Hypomanic Checklist – 16 items, *FIBSER* Frequency, Intensity and Burden of Side Effects Ratings, *PRISE* Patient Rated Inventory of Side Effects, *EQ-5D* EuroQol-5D health index, *THINC-it* THINC-it tool for cognitive dysfunction in Major Depressive Disorder, *DSCT* Digit Symbol Coding Test, *WSAS* Work and Social Adjustment Scale, *ASRM* Altman Self-Rating Mania Scale, *VAS* Visual Analogue Scale, *GAD-7* Generalised Anxiety Disorder questionnaire – 7 items, *SAPAS* Standard Assessment of Personality: abbreviated Scale, *CSRI* Client Service Receipt Inventory, *FBC* Full blood count, *U&Es* Urea, electrolytes and creatinine, *LFTS* Liver function tests, *TFT* Thyroid function tests

^a^ Optional and/or collected in a subset of participants

^b^ Measures solely for tertiary and ancillary analyses

### Screening visit

All participants must provide written informed consent at the start of the screening assessment. Consent may be taken by a trial clinician, or delegated non-clinical researcher with trust approval. Confirmation of eligibility will be signed off by a trial clinician at the end of this assessment.

### Baseline, weekly monitoring and follow up visits

If the baseline assessment is more than 7 days after the screening visit, the screening assessments (excluding demographic information) will be repeated to ensure that the participant still meets the required eligibility criteria. Randomisation will be conducted at Week/Time Point 0, which is the day of the baseline visit. Follow-up assessments with trial researchers will be conducted at 8 (+ − 1) weeks, 26 (+ − 2) weeks, and 52 (+ − 2) weeks post-randomisation.

Patients will also be asked to complete three questionnaires weekly via the True Colours system (https://truecolours.nhs.uk): the QIDS-SR, WSAS, and study specific questions about medication status. True Colours is an online monitoring tool on which questionnaires can be completed via email, text, or by logging on to the website. For patients without internet access, a paper version can be provided. Automated reminders will be sent to participants on a day and time of their choosing.

Optional study blood tests will be conducted at baseline, 8 and 52 week visits (agreement to clinical blood tests throughout the trial duration is not optional). Optional blood tests include samples for genetic and cytokine analysis, along with hair samples to assess cortisol levels, and saliva samples for genetic analysis where blood samples are not provided. This will be done in collaboration with the Biomedical Research Council (BRC) BioResource. Further optional measures include a qualitative interview exploring patient experience of True Colours as a mood monitoring tool (conducted at one follow up appointment) and the THINC-it tool for cognitive function [46].

One section of the CSRI collects data about the patient’s principal formal or informal carer. Where applicable, the carer can complete this questionnaire following their provision of written informed consent. Where this is not possible, responses from the patient will be accepted.

At 4 weeks post-randomisation, participants will receive a phone call from a trial researcher to determine the date they began taking their allocated medication. If applicable, the reason the patient has not commenced their medication will be recorded and this question will be repeated at follow-up visits to determine if a patient has subsequently begun the allocated medication.

All study data will be stored on a custom-designed, online data entry system which is compliant with Good Clinical Practice guidelines, and created and maintained by King’s Clinical Trials Unit (InferMed MACRO version 4.0). The system is programmed to perform validation checks for data quality purposes (e.g. range checks to avoid data entry errors) and flags out of range or missing data for monitoring purposes. It also has a built in audit trail to aid data monitoring.

### Statistical methods for analysing primary and secondary outcomes

A full statistical analysis plan will be drawn up prior to completion of data collection. The main analysis will follow an intention to treat (ITT) principle, whereby patient data are analysed by treatment group, regardless of the medication status of patients throughout the follow-up period.

Participant flow will be presented in a CONSORT diagram. For analysis of the listed primary and secondary outcomes, missing baseline data will be filled in using imputation [47]. Models will covary for randomisation stratification factors throughout. For the self-report QIDS-SR score a linear mixed model, covarying by baseline score, will be used for the estimation of the mean treatment difference and 95% confidence interval (CI) over the course of the trial. Full information maximum likelihood methods will be used for analysis, which will account for missing data under the missing at random assumption. Error distributions will be checked using Q-Q plots. Time to discontinuation will be calculated from first prescription date to treatment discontinuation, and will be analysed using Kaplan-Meier methods and Cox regression models, providing the hazard ratio for treatment discontinuation in lithium versus quetiapine, and associated 95% CI.

Continuous secondary outcomes measured over time will use generalised linear models appropriate to the variable distribution covarying by baseline where applicable, and using mixed models where there are repeated measurements. Adjustment for multiple testing will be presented [48].

For measures where missingness is above 5% and where the absence of repeated measurement gives less confidence in the adequacy of the missing data properties of maximum likelihood, we may undertake a sensitivity analysis within a multiple imputation framework [49].

### Power calculation

Based on our power calculation and superiority design, we require 276 patients to be randomised. When considering the sample size required, we have considered both the primary outcome measures of time to all cause treatment discontinuation and longitudinal symptom severity as assessed by QIDS-SR scores. This sample size was calculated based on the following assumptions:

- *Treatment discontinuation:* estimated 20% discontinuation from treatments by 8 week follow up visit (as per Bauer et al., 2013) [20]), and 50% treatment discontinuation for lithium and 30% for quetiapine by 52 weeks.
- *Missing data:* 40% missingness for each of the post-randomisation QIDS-SR scores was allowed for. While the missingness has been assumed independent over time, the QIDS-SR scores are assumed uniformly correlated with a correlation of 0.6 [47]. We have allowed the discontinuation status at follow-up to be unknown for 10% of the sample.
- *Effect size:* The minimum clinically significant difference for outcomes in depression treatment is widely taken to be 3 points on the HDRS [3, 50]. This corresponds to an effect size of 0.38 between treatments. We wish to see a difference of this effect size in the QIDS-SR score sustained over the duration of the trial and so will estimate the effect as an area under the curve.
- *Power*: All power calculations are for two-tailed tests and alpha = 0.05.

With a sample size of 276 at baseline and 10% loss to follow up, we expect 248.4 patients to complete the trial. The Stata stpower command gave 90% power using a logrank test to detect a difference in the time to discontinuation with 50% (lithium) and 70% (quetiapine) as the proportions of participants remaining on assigned treatment. This determined the lower limit of the sample size. Applying this sample size to the self-report QIDS-SR data we also needed to account for the likely haphazard nature of missing data points at each assessment time point. We used simulation and the non-central chi-square method. Using 1000 samples, a simple random intercept model covarying for baseline, a time dummy variable and with a single average combined treatment effect, this number of participants will provide 99.7% power to detect an effect size of 0.38 (with 1 degree of freedom, χ^2^ = 3.84, and non-centrality parameter = 20.86).

### Monitoring

There is no planned interim analysis and no specific “stopping rules” for the study. We have an independent Data Monitoring and Ethics Committee and an independent Trial Steering Committee. SAEs will be reported to the relevant bodies for all patients throughout their involvement in the trial. Clinicians have the right to discontinue the study drug in the event of identified contraindications, inter-current illness, pregnancy, adverse drug reactions, adverse events, protocol violations, administrative causes, or for other reasons. We will adhere to NHS confidentiality practice, and to the Research Governance Framework in monitoring, auditing and managing the research.

## Discussion

This protocol paper describes a pragmatic randomised clinical trial designed to determine whether the decision to prescribe quetiapine is more clinically and cost effective than lithium for patients with TRD over 12 months. The chronicity of TRD, in which patients often fluctuate between states of remission and relapse [51], highlights not only the importance of this research question, but also the implementation of a 12 month follow up period. As discussed, there have been no direct head-to-head comparisons of these treatments with a follow up period of greater than six weeks. This is insufficient given that up to 80% of patients with TRD relapse within 12 months of responding to treatment [8, 20]. Our choice of primary outcome, a longitudinal measure of depression severity (QIDS-SR), measured weekly over 12 months via the True Colours system (www.truecolours.nhs.uk), recognises the highly fluctuating nature of this disorder. True Colours has been used to assess mood in prior research studies with success [52] and facilitates the monitoring of all cause treatment discontinuation across the full duration of the trial.

The benefits of determining whether one of these recommended and widely prescribed medications is more effective for patients with TRD are clear. However the results of this study should also positively impact secondary care mental health teams and the wider NHS by helping to minimise relapse rates and in doing so alleviate some of the financial and time burden placed on mental health services. If there is no significant difference between lithium and quetiapine in the primary outcomes of clinical effectiveness, the cost-effectiveness analysis, which takes into account the different monitoring requirements for these medications, may benefit health bodies by informing them of the most cost-effective of these two treatment options. In addition, our further analyses may contribute to the growing body of research examining predictors of treatment response in depression, and the development of personalised mental health care.

The potential impact of this trial is maximised by its pragmatic design, which ensures that the results will be directly applicable to real-world clinical practice. The PRECIS-2 tool [51] was used to assess how far we have achieved this and what elements of the design are most pragmatic. A key pragmatic strength is our choice of primary outcomes (the effectiveness and tolerability of the treatments) which are directly applicable to, and can clearly be understood by, the patient group. Additionally, our broad inclusion criteria and minimal exclusion criteria enable us to capture as much variation in the TRD population as possible and include the majority of patients that are likely to receive such an augmentation therapy in routine clinical practice. Examples of this are the inclusion of patients demonstrating suicidal ideation, as this group is often excluded from clinical trials, and the decision not to include an upper age limit (commonly set at 65 years for TRD clinical research) improving the applicability of our results to older patients [3]. Furthermore, alongside the recruitment of patients from secondary care settings, where lithium or atypical antipsychotic augmentation is commonly initiated, we are also recruiting patients from primary care. In doing so we are ensuring that our samples reflect the range of patients that should be considered for these two augmentation therapies according to best practice guidelines.

The pragmatic design of the trial is also reflected in our decision to not dictate a specific dosing or monitoring schedule. As discussed, clinicians are provided with best-practice dosing and monitoring guidelines, but are free to prescribe, monitor and discontinue the trial medication as they find clinically appropriate.

The PRECIS-2 tool also highlighted some necessary limitations to the trial’s pragmatism; primarily the large number of measures conducted during the follow up period. As with all pragmatically designed trials, there must be a balance between the need for adequate data collection and the additional burden this places on participants, beyond what would be experienced in normal clinical practice. However, all chosen measures were deemed necessary by the study team to ensure that the results of the trial aid the development of a modified and evidence based treatment pathway for TRD, and contribute to the improvement of treatment outcomes in this patient group. Conducting a thorough psychiatric history assessment to determine TRD staging and depression characteristics, assessing personality traits relating to refractoriness, evaluating mixed and atypical depressive symptomatology, and collecting biological samples to examine genetic and inflammatory markers and changes in physical health, will allow for a thorough comparison between the two treatments. In order to minimise patient dropout, some of the measures associated with tertiary or ancillary outcomes only have been made optional for participants. Additionally, information will be gathered from medical records or at future follow up visits where possible should a patient fail to attend or complete an assessment.

The LQD study is a large scale trial designed to build on evidence reported by previous short term comparisons of lithium and quetiapine augmentation in patients with TRD [21, 22]. In doing so, we hope to definitively determine which of these two augmentation treatments (if either) should be preferential over the other, improving patient outcomes and minimising the burden that TRD places on clinical resources.

## Trial status

Recruitment is currently ongoing at 4 sites (SLaM, NTW, OHT, and TEWV) and is set to continue until February 2019. It is anticipated that the trial will end in June 2020.

## Abbreviations

ASRM: Altman Self-Rating Mania Scale
BAP: British Association for Psychopharmacology
BNF: British National Formulary
CGI: Clinical Global Impressions
CI: Confidence interval
CSRI: Client Service Receipt Inventory
DSCT: Digit Symbol Coding Test
DSM-V: Diagnostic and Statistical Manual of Mental Disorders – Fifth Edition
ECG: Electrocardiogram
EQ-5D: EuroQol-5D health index
FBC: Full blood count
FIBSER: Frequency, Intensity and Burden of Side Effects Ratings
GAD-7: Generalised Anxiety Disorder questionnaire – 7 items
HCL-16: Hypomanic Checklist – 16 items
HDRS-17: Hamilton Depression Rating Scale – 17 items
IDS-C: Inventory of Depressive Symptomatology – Clinician Rated
ITT: Intention to treat
LFTS: Liver function tests
LQD: Lithium versus Quetiapine in Depression
MÅDRS: Montgomery-Åsberg Depression Rating Scale
MARS-5: Medication Adherence Report Scale – 5 items
MDD: Major depressive disorder
MINI 7.0: Mini International Neuropsychiatric Interview, Version 7.0 for DSM-5
MPG: Maudsley Prescribing Guidelines
MSM: Maudsley Staging Method
NHS: National Health Service
NICE: National Institute for Health and Care Excellence
NIHR: National Institute of Health Research
NTW: Northumberland, Tyne and Wear NHS Foundation Trust
OHT: Oxford Health NHS Foundation Trust
PRISE: Patient Rated Inventory of Side Effects
QIDS-SR: Quick Inventory of Depressive Symptomatology – Self-Rated
quetiapine XR: quetiapine extended release
SAEs: Serious Adverse Events
SAPAS: Standard Assessment of Personality: abbreviated Scale
SLaM: South London and Maudsley NHS Foundation Trust
SPIRIT: Standard Protocol Items: Recommendations for Interventional Trials
SSRI: Selective serotonin reuptake inhibitor
TEWV: Tees, Esk and Wear Valleys NHS Foundation Trust
TFT: Thyroid function tests
THINC-it: THINC-it tool for cognitive dysfunction in major depressive disorder
TRD: Treatment resistant depression
TSQM: Treatment Satisfaction Questionnaire for Medication
U&Es: Urea, electrolytes and creatinine
VAS: Visual Analogue Scale
WSAS: Work and Social Adjustment Scale
